# Fused Deposition Modeling 3D Printing in Oral and Maxillofacial Surgery: Problems and Solutions

**DOI:** 10.7759/cureus.28906

**Published:** 2022-09-07

**Authors:** Takashi Kamio, Takeshi Onda

**Affiliations:** 1 Oral and Maxillofacial Radiology, The Nippon Dental University, Tokyo, JPN; 2 Oral and Maxillofacial Surgery, Tokyo Dental College, Chiba, JPN

**Keywords:** simulation model, patient-specific model, oral and maxillofacial surgery, fdm, ‎3d printing

## Abstract

Three-dimensional (3D) printing technology in medicine is one of the new and innovative technology for fabricating 3D models of complex anatomical structures that can be observed both visually and haptically. Patient-specific 3D models fabricated through this process are currently being used for various purposes, including surgical simulation, training, and medical education. Most of the personal use/low-end desktop 3D printers that are becoming widespread are fused deposition modeling (FDM) 3D printers. Compared to professional/high-end 3D printers, the price of the personal use/low-end desktop FDM 3D printer itself, filament, and running costs are lower; it can lower the economic bottleneck for introducing 3D printing technology into clinical practice, such as surgical simulation. With a desktop FDM 3D printer and a general-purpose PC, anyone can now rapidly fabricate 3D models on their own without having to rely on 3D printing labs and specialized technicians. However, it is also true that FDM 3D printers, due to their mechanical characteristics, encounter many difficulties and problems that emerge during the 3D printing process. Knowledge, know-how, and tips about FDM 3D printers have been introduced in various media, and it has become easy to know about them worldwide via the Internet. However, there has been no comprehensive technical review to date to produce osseous 3D models for use in oral and maxillofacial surgery. In this report, to create 3D models according to the characteristics of maxillofacial and oral surgery, we enable surgeons themselves to create 3D models smoothly by presenting ideas for CT scanning, points to note when exporting Digital Imaging and Communications in Medicine (DICOM) image data, how to create optimal stereolithography (STL) models, and problems and solutions for 3D printing.

## Introduction

Since the late 1990s, anatomical and life-like three-dimensional (3D) models have had a definite place in cranio-maxillofacial surgery [1,2]. More recently, advances in 3D printing technology and the progress and generalization of hardware and software have made it easier to fabricate full-scale 3D models of various organs and obtain them. Currently, the field of oral and maxillofacial surgery is also increasing the use of patient-specific anatomical 3D models for surgical simulation, training, and medical education [3-5]. The feature of the 3D model is the ability to visualize delicate and complex anatomical structures and to observe and touch them from any direction. These can provide a three-dimensional understanding of anatomical structures for the surgeon, and the use of simulation can help improve the predictability of surgery, which in turn contributes to surgical safety.

Around 2010, at about the same time as the worldwide 3D printer boom, our team developed a 3D printing lab for use in the field of oral and maxillofacial surgery [6]. This lab can fabricate low-cost 3D models with short delivery times, without the need for specialized 3D printing labs or expert technicians. We have demonstrated that in orthognathic surgery, surgical simulation using fabricated osseous 3D models increases the surgeon's surgical certainty, decreases intraoperative blood loss, and shortens operative time [7]. These 3D models are produced with commercially available desktop fused deposition modeling (FDM) 3D printers. Compared to high-end, expensive professional 3D printers, these printers are very inexpensive, yet can produce osseous 3D models sufficient for clinical use [8,9].

To fabricate osseous 3D models, it is necessary to know how to create an optimal 3D computer-aided design (CAD) model data and to understand the fabrication characteristics of FDM 3D printers. However, until now, there have been no reports or manuals that specifically cover the series of processes in the 3D model fabrication based on the specifics of oral and maxillofacial surgery, and it has required a lot of time to learn them. This report presents solutions learned from many 3D printing failures and describes strategies for FDM 3D printing with reduced failures.The goal is to make this cost-effective 3D printing technology available to all fields that require osseous 3D models.

## Technical report

Fundamentals and vocabulary

One problem in describing the fabrication of 3D models is that it has been very difficult to unify the various terms used in their associated jargon. This is because the terminology varies from literature to literature, from manufacturer to manufacturer, and from user to user. Most of the 3D printing terms used in this report are based on the recommendations by the International Organization for Standardization (ISO/ASTM 52900:2021) [10]. Here, "3D printing" indicates the production of 3D models using a 3D printer; and "fabrication" indicates the entire series of processes to create that 3D model, including segmentation and binarization of Digital Imaging and Communications in Medicine (DICOM) images on the medical imaging software packages, 3D printing with the 3D printer, removal of support structures, and final finishing.

**Figure 1:**
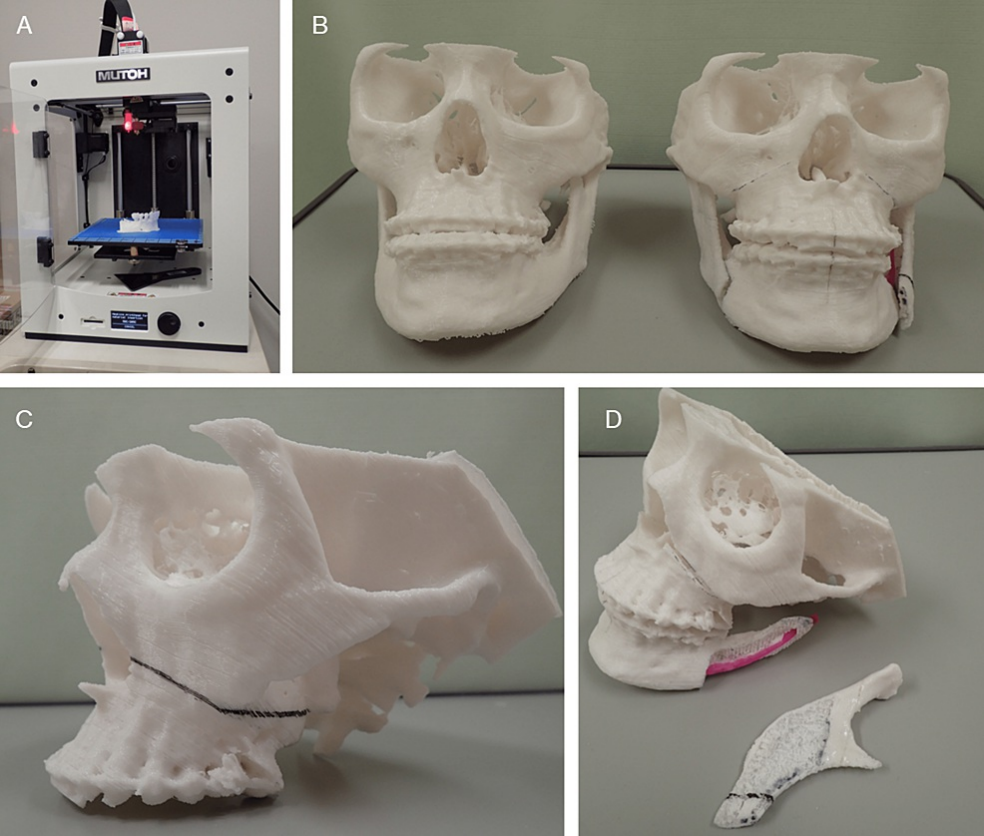
3D models for surgical simulation of Le Fort I osteotomy and sagittal splitting ramus osteotomy in oral and maxillofacial surgery. Both models were 3D printed with a laminating pitch of 0.3 mm; the 3D printing time for one 3D model was approximately 18 h. (A) FDM 3D printer "Value3DMagiX MF-800". (B) Fabrication of two 3D models facilitates understanding of pre- and post-operative change. These are useful for providing patients with pre-operative explanations. (C, D) Surgical simulation with 3D models allows a "hands-on" understanding of the amount and direction of bone movement and areas of interference between bone segments.

Stereolithography (STL), also referred to as "Standard Triangle Language" or "Standard Tessellation Language" is a commonly used data format (.stl) that represents the surface of an object as a mesh of small triangles. It has a long history and is the de facto standard file format used in fields such as 3D CAD, rapid prototyping, and more recently, 3D printing. In this report, STL format data will be called "STL data," and the polygonal mesh models created based on these data will be called "STL model". In the process of converting a DICOM image into a 3D printable data file, it is first necessary to create intermediate data. These data serve as an intermediary, which interacts with data produced by a range of 3D CAD software packages (synonymous with STL model data in this report). The format of the exported file needed to proceed to the next step depends on the medical imaging software (DICOM segmentation software) and/or 3D CAD software (polygon editing software) used. Many of them have "saved as STL file" or "exporting to STL" commands, however, and it is common for it to be exported as a data file in STL format. Although the FDM mechanism is currently also called the fused filament fabrication (FFF) mechanism, this report will refer to it as FDM, following the catalog of 3D printer manufacturers. Figure 1 shows the 3D models we fabricate and use in clinical oral and maxillofacial surgery.

Mechanism, features, and the fabrication process of the FDM 3D printer

Desktop FDM 3D printers, which are classified as personal and low-end, offer great advantages in terms of compactness, maintenance, and running cost. Moreover, FDM 3D printers are also environmentally friendly as they do not require any special materials such as resin solutions, which other types of 3D printers do. Furthermore, in printing FDM 3D models, the filaments are melted at approximately 200 degrees Celsius. They can be sterilized with high-pressure steam, as is the case with many medical devices. This means that they can be brought into the operating room.

**Figure 2:**
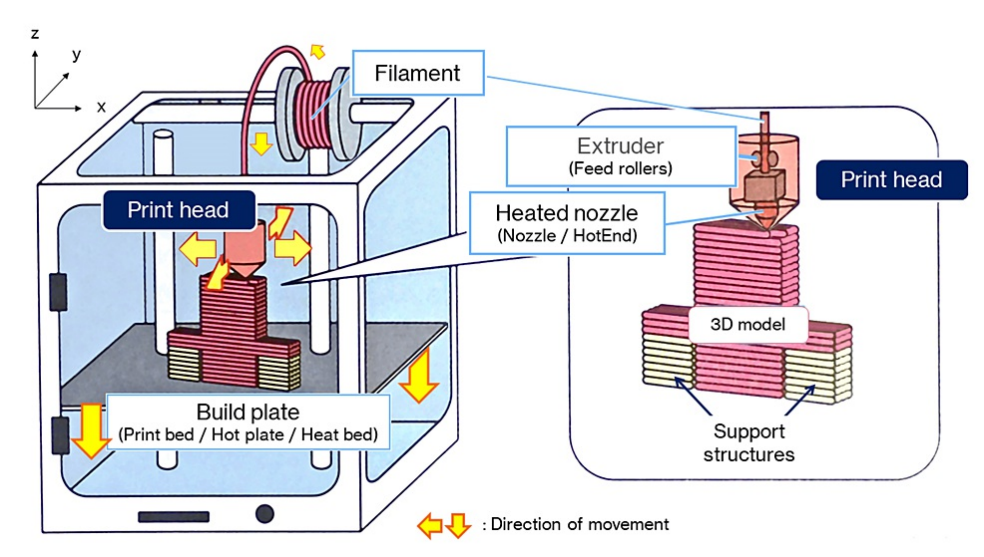
Mechanisms of the FDM 3D printer. The FDM printing process begins by loading a spool of thermoplastic resin filament into a 3D printer. Once the nozzle reaches the target temperature, the filament is fed into the print head, where it is melted. The print head is mounted on a 3-axis system and can be moved in x-, y-, and z-directions. The molten filament is extruded into thin wadding and laminated layer-by-layer in place, where it is cooled and solidified. Once one layer is completed, the build plate is moved down and the new layer laminated. This process is repeated until the 3D model is complete.

Figure 2 shows the mechanism of the FDM 3D printer. The melted resin is extruded through a nozzle to form layers in single strokes, a process resembling that seen in a soft-serve ice cream machine. The time taken for FDM printing increases with the size and/or many 3D models required. Another factor to be considered is warpage due to heat-induced shrinkage, which increases with the size of the model required. The standard filaments used in the FDM 3D printers are polylactic acid (PLA) and acrylonitrile butadiene styrene (ABS). Our team uses PLA because it has a stiffness similar to that of cortical bone and is suitable for osteotomy simulation, as well as for ease of availability and cost. One problem is that all these filaments change over time, becoming brittle as they absorb moisture. Under normal conditions of use and storage, however, it takes several months before they are no longer usable (Note: this does not mean that they are unusable, but rather that they frequently break or plug up in printing). Therefore, this should not pose too much of a problem.

**Figure 3:**
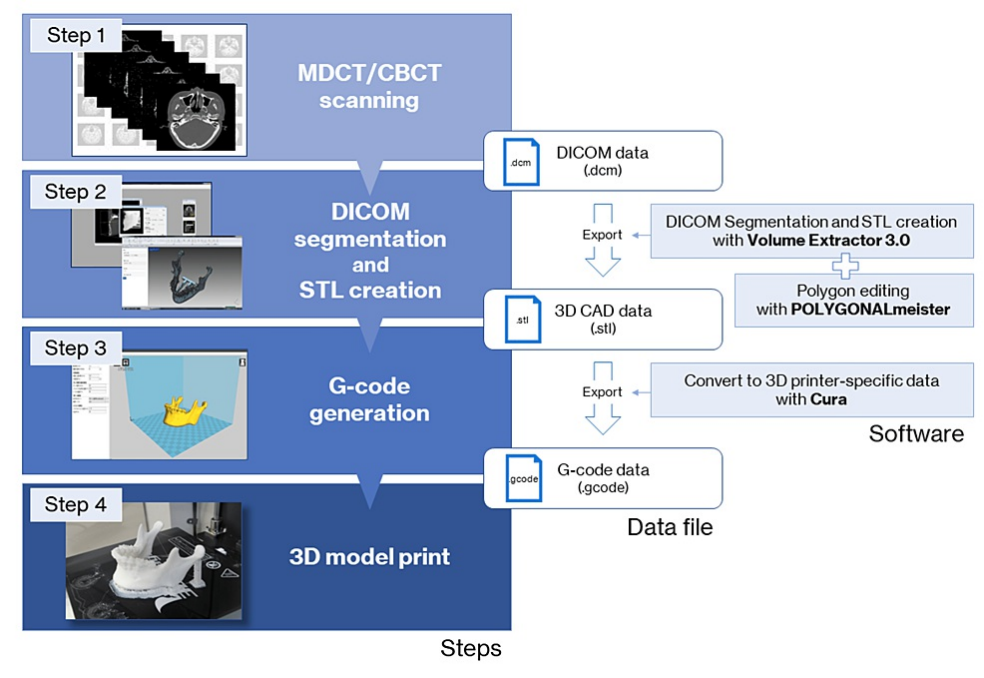
The fabrication process workflow for the 3D model with an FDM 3D printer. (Step 1) Perform CT scan and export data as a DICOM file. (Step 2) Create a 3D CAD model in STL format. (Step 3) Generate G-code. (Step 4) 3D printing with FDM 3D printer. 3D: Three-dimensional, CAD: Computer-aided design, CT: Computed tomography, CBCT: Cone-beam CT, DICOM: Digital Imaging and Communications in Medicine (file format), FDM: Fused deposition modeling, MDCT: Multidetector row computed tomography, STL: Stereolithography (file format). Cura: Slicing software package, G-code: Used mainly in computer-aided manufacturing to control automated machine tool tools, POLYGONALmeister: Polygon editing software package, Volume Extractor 3.0: STL conversion software package.

The 3D model fabrication process workflow is shown in Figure 3. The data exported from medical imaging modalities such as multidetector-row computed tomography (MDCT) and limited cone-beam computed tomography (CBCT) in the form of DICOM files cannot be directly used to fabricate a 3D model. To fabricate a 3D model with a 3D printer, it is first necessary to prepare the data that will serve as the blueprint for the 3D model. Optical 3D scanners are often used for the acquisition of STL data required for the manufacture of handmade products. In the medical field, DICOM images obtained from medical imaging modalities, mainly MDCT, are converted to STL data and prepared after binarization, setting of regions of interest, and other processing. In the binarization process, which determines whether to 3D print or not, the threshold value of voxel intensity (equivalent to the Hounsfield unit in MDCT) greatly affects the geometric shapes of the STL model. The operator of the medical image processing software (DICOM segmentation software) determines the binarization threshold, which differs from operator to operator. If the threshold is too high, the replication of thin bones will be poor. Alternatively, a low threshold value would result in a low-quality STL model with a rough surface because it would include a voxel intensity as high as that of the surrounding soft tissue in addition to metal artifacts.

The created STL model is exported to the slicing software (also known as a slicer, this software generates data (G-code), which mediates between the 3D model and the 3D printer). G-code is the most widely used computer numerical control (CNC) including FDM 3D printers programming language in the industry. It is generated by the slicing software and contains various parameters for operating the 3D printer, such as printing orientation (direction of lamination), laminating pitch, infill density, supporting properties (shape, infill pattern, filling density of supports, overhang angle, z-distance), the temperature of the heated nozzle and build plate, and print speed. After the 3D printing is completed, the support structures are removed from the 3D model, and the 3D model is provided with finishing touch-ups such as deburring and trimming.

Dimensional accuracy of osseous FDM 3D models

To investigate the dimensional accuracy, four mandibular 3D models with different laminating pitches were fabricated, and the STL model created by reverse scanning each model was compared to the master STL model. The shape error was measured by comparing the shape with the master STL model and the STL model created by reverse scanning. Table 1 and Table 2 show the 3D printing parameters and the reverse scanning parameters of the MDCT. The FDM 3D printer used for the fabrication was Value3DMagiX MF-800 (MUTOH Industries Ltd., Tokyo, Japan). A 64-slice MDCT Aquilion (Canon Medical Systems, Otawara, Japan) was used for reverse scanning. 3D CAD comparison and inspection software package SpGauge 2014.1 (Armonicos Co., Ltd., Hamamatsu, Japan) was used to visualize and measure shape errors.

**Table 1:** 3D printer and print parameters used in the fabrication of 3D models.

3D Printing parameters	
3D printer	Desktop FDM 3D printer - Value3DMagiX MF-800 (MUTOH)
Filament	PLA 1.75mm Filament - PolyLite PLA (Polymaker)
Print speed	48 mm/s
Infill density/Infill pattern	33 % / Grid-shaped infill
Flow rate	100 %
With/without support	With raft-shaped supports
Heated nozzle temperature	210°C

**Table 2:** Reverse scanning parameters of the multidetector-row computed tomography (MDCT).

Scanner and scanning parameters	
Scanner	64-slice MDCT - Aquilion 64 (Canon Medical Systems)
Tube voltage	120 kV, 50 mAs
Slice thickness	0.5 mm
Field of view (FOV)	240 mm
Matrix	512 × 512
Convolution kernel	FC81 (bone sharp)

**Figure 4:**
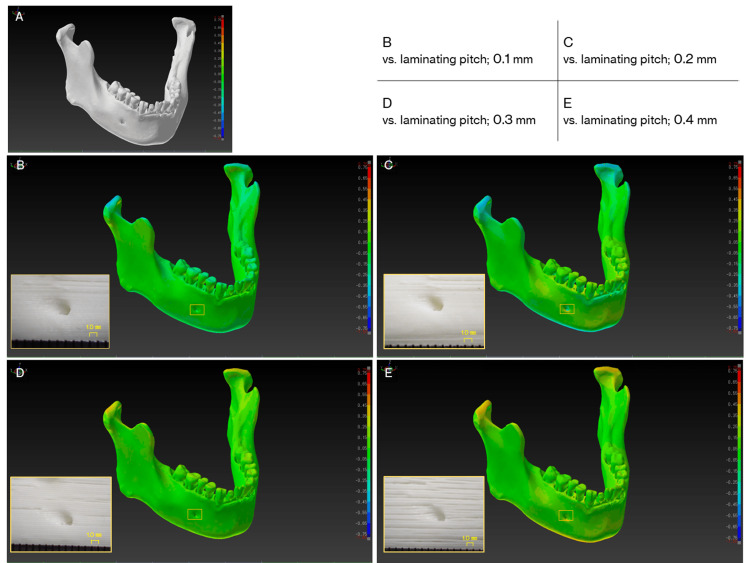
Visualization of dimensional errors (signed differences) for each 3D computer-aided design (CAD) model. The warm colors show expansion from the master 3D CAD model in stereolithography (STL) format, while the cold colors show shrinkage. In this color map, the maximum value for red represents 0.75 mm and the minimum value for blue represents -0.75 mm. (A) The master STL model. (B-E) Visualization of shape errors between the master STL model and the reverse scan STL model, and close-up view of the 3D model surface of the mental foramen area at each laminating pitch (squares).

The polygon count of the master STL model and each STL model after reverse scanning was about 600,000 and the file size was 30 megabytes. Table 3 and Figure 4 show the results of the 3D model after fabrication, with a color map image and a close-up view of the 3D model surface after the master STL model and reverse scanned STL model was superimposed. The results showed that increasing the laminating pitch reduced the 3D printing time, but the surface of the 3D model became rougher and streaky steps were more noticeable. In comparison with the master STL model, some shape errors were observed in the z-direction (condyle - inferior margin of the mandible direction), but they were almost within plus or minus 0.5 mm.

**Table 3:** Results of 3D printing and comparison with master stereolithography (STL) model. ^a^: Weight without supports. ^b^: Actual 3D model printing cost excluding supports.

Results of 3D printing				
Laminating pitch	0.1 mm	0.2 mm	0.3 mm	0.4 mm
3D Print time	18 h 40 m	9 h 35 m	6 h 43 m	5 h 31 m
Weight ^a^	79 g	79 g	78 g	78 g
Fabrication cost ^b^	2.05 USD	2.05 USD	2.02 USD	2.02 USD
Comparison with master STL model				
Mean absolute shape error	0.04 mm	0.02 mm	0.01 mm	0.02 mm
Maximum shape error	0.2 mm	0.6 mm	0.7 mm	0.6 mm
Minimum shape error	-0.2 mm	-0.5 mm	-0.8 mm	-0.6 mm
Standard deviation	0.05	0.17	0.08	0.11

## Discussion

3D models reproduce the complex structures of the teeth and jaws (e.g., foramen, cavities, processes, ridges, thin bone), allowing us to confirm bone thickness, cavity depth, and blind spaces that are difficult to see directly. It can also be used to simulate realistic surgery. Many young surgeons who want to improve their surgical skills must be trained in practice models. However, obtaining these models has generally required high costs. For them, inexpensive, patient-specific 3D models would be a great help [11,12].

FDM 3D printers are generally used for personal use, such as for hobbies and crafts. When they are used to fabricate anatomical 3D models and for medical applications such as surgical simulations, it is natural to be concerned about their 3D printing accuracy. In 2017, George et al. [13] investigated the accuracy and reproducibility of 3D printed medical models, in which they reported that the advantage of FDM 3D printers is low cost, but that the spatial resolution is lower than that with other modalities. There are no correct CT scanning parameters, STL model creation parameters, or 3D printing parameters for fabricating 3D models. Therefore, the protocol adopted in this report follows our previous reports [6,14] and the report by Mitsouras et al. [15]. A slight dimensional error occurred between the master STL model and the STL model due to the amount of laminating pitch, but all were within plus or minus 0.5 mm. The reason for the slight error in dimensional shape was assumed to be due to the individuality of the software used to convert the DICOM image to STL data (derived from the binarization algorithm implemented in the software) and/or the deformation of the filament during the 3D printing process. However, the error is the size of one or two voxels of an MDCT image and is the limit of the spatial resolution of the MDCT used in this study. These results show that dimensional errors caused by differences in laminating pitch do not significantly affect practical operations such as surgical simulations.

**Figure 5:**
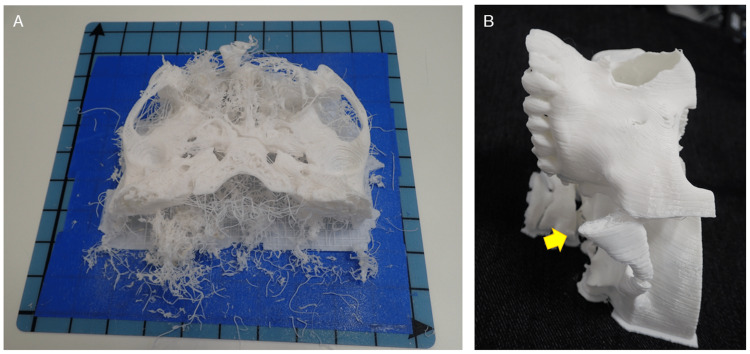
Examples of 3D printing failures. (A) Failure is caused by a combination of factors. It is not easy to determine the cause of such failure. (B) Failure of 3D model fabrication. The zygomatic arch was too narrow to bear the weight of subsequent filament and therefore tilted (arrow).

Using the FDM 3D printer is a constant struggle against the instability of the 3D printing process. We have experienced numerous 3D printing failures (Figure 5). They are frequently the result of a combination of two or three interrelated factors, and finding a solution to them is difficult.

**Figure 6:**
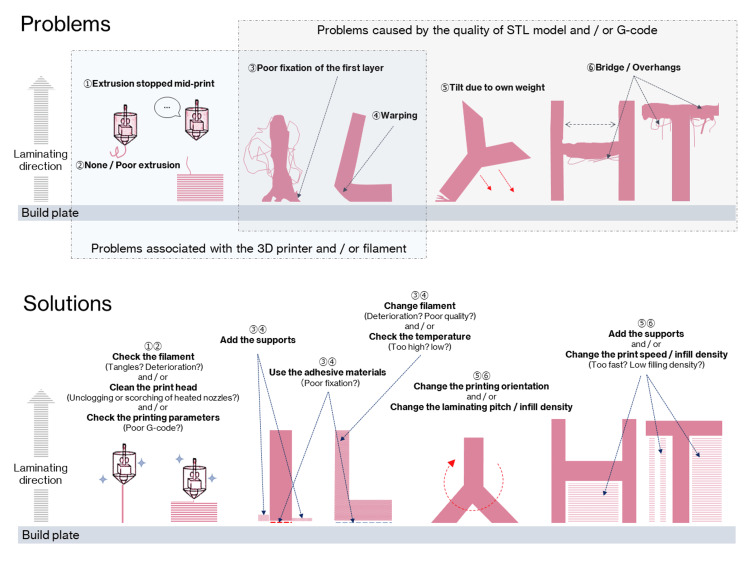
Fused deposition modeling (FDM) 3D printing problems and solutions. Circled numbers in problems correspond to circled numbers in solutions.

**Figure 7:**
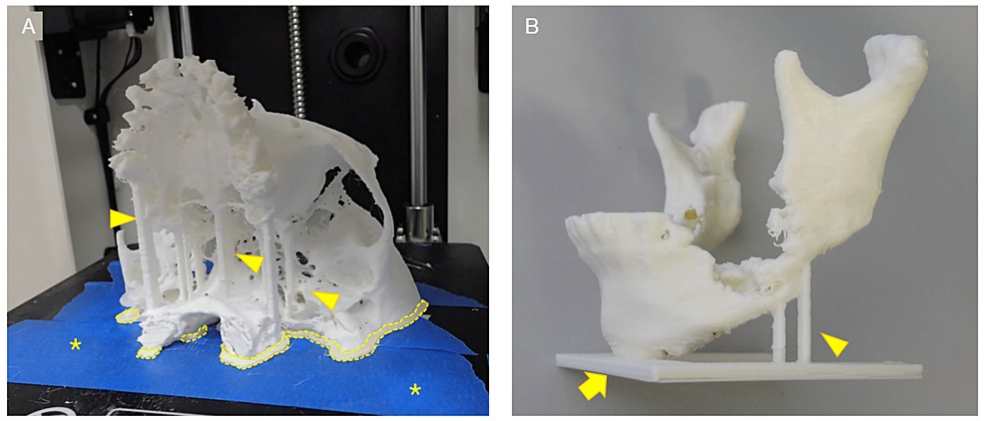
Various types of support. (A) Optional columnar support added by the operator (arrowheads) and raft-shaped marginal supports generated semi-automatically by slicing software (dotted line). The raft-shaped support increases the area of fixation between the 3D model and the build plate and prevents the 3D model from displacing during 3D printing. Masking tape (blue area marked with *) was applied in the hope of improving fixation between the 3D model and the build plate. (B) Support for column-shaped supports (arrowheads) and table-shaped (arrow). Both were placed during the creation of the STL model.

**Figure 8:**
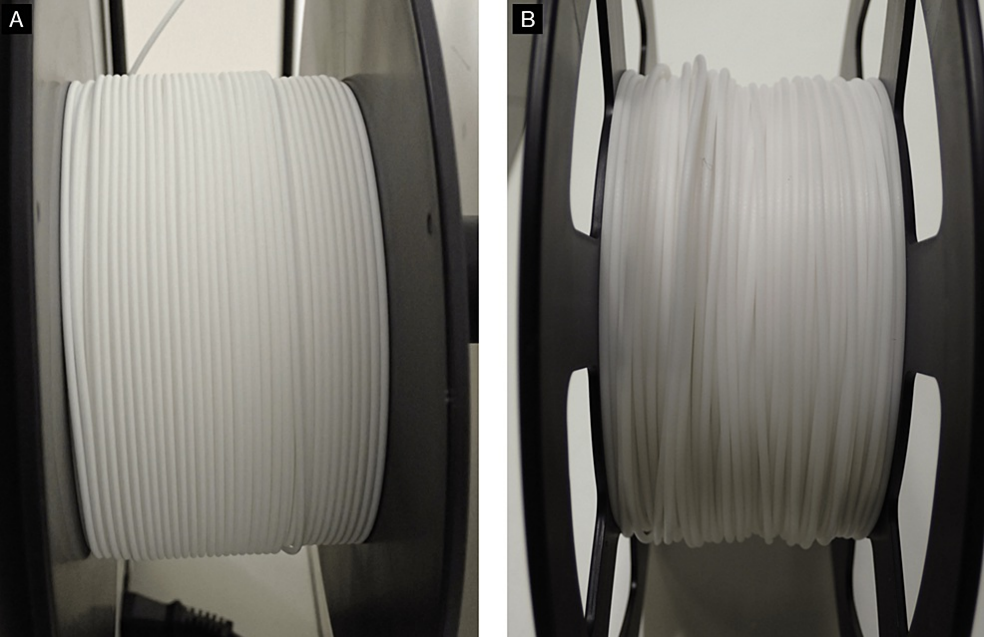
Differences in filament winding by the manufacturer. (A) Filament neatly wound on the reel. (B) Uneven winding can lead to filament tangling during 3D printing.

Figure 6 shows some problems that can be encountered in FDM 3D printing and their suggested solutions. Here, these problems have been divided into two major categories: those caused by poor quality STL models or G-code; and those caused by the 3D printer or filament. To avoid 3D printing failures and achieve smooth 3D printing, it is first important to properly create the STL model. As a solution to 3D printing failures derived from the quality of the STL model, during STL model creation, thin areas of bone that are expected to be "perforated" by binarization, such as the maxillary sinus wall, condylar, and anterior ramus margin, are compensated by filling or adding columnar supports (Figure 7). Table-shaped supports are also sometimes added, for parts that have little contact area with the build plate. A raft-, brim-, or skirt-shaped extra marginal supportive layers may be used to improve stability. As a solution to failures caused by 3D printers and filaments, in our experience, the most common problem is the failure to fix the first layer. Problems caused by the failure of the first layer are commonly referred to as "First layer problems" or "First layer issues". This is arguably the biggest problem bothering all FDM 3D printer users [16]. Filaments are prone to deformation due to prolonged exposure to heat from the build plate during 3D printing, with ABS filaments being particularly prone to this. Therefore, lowering the temperature of the build plate (Note: with some FDM 3D printers, the temperature of the build plate can be adjusted but not all) or using auxiliary materials such as masking tape, hairspray, or glue sticks can improve fixation and reduce filament warpage. Care must also be taken with the quality of the filament itself. Some filaments are not wounded neatly and can become entangled during the 3D printing process, increasing the risk of failure (Figure 8). Additionally, the properties of the same brand sometimes differ among production lots. The 3D printing parameters differ from the quality of the 3D printer and the filament used, but in any case, if they are improper, it will directly lead to the failure of the 3D print. In other words, the quality of the G-code determines the outcome. In the current situation, where 3D printing operator ingenuity is required, the best parameters/settings for each 3D printer and filament quality must be determined based on their own experience.

**Figure 9:**
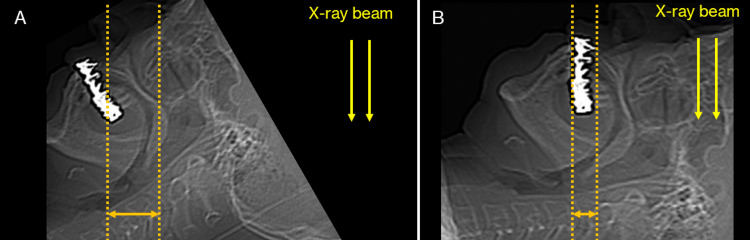
An approach to reduce the range of metal artifacts. The number of metal artifacts differs depending on the positioning of the head (jaw position). These schematics were produced using scout images from CT scanning and were not scanned in the position shown in Figure A. (A) Metal artifacts appear over a large range. (B) By changing the position, the range in which metal artifacts appear can be narrowed.

**Figure 10:**
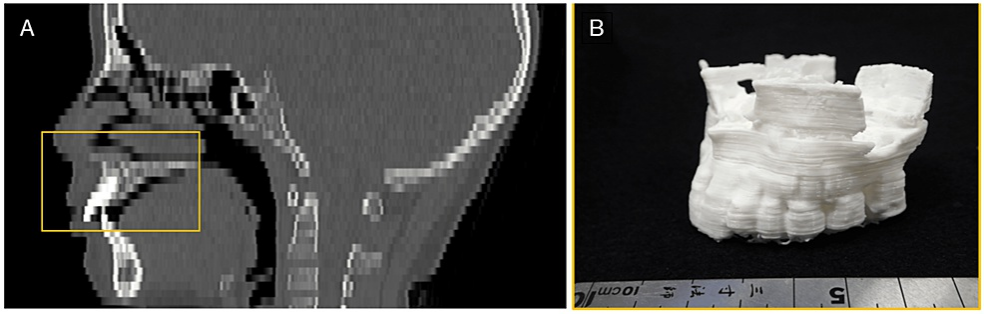
An example of 3D modeling from DICOM image data with too-thick slices. An example of a maxilla (square) that had to be segmented and 3D printed, even though the quality of the original images for STL model creation was poor. (A) CT median sagittal image of the head. A thick slice thickness results in stepped edges and poor reproduction of detail. (B) 3D model fabricated from these images. 3D CAD software was used to edit polygons, including smoothing the STL model, but there were limitations, resulting in a rough 3D model. CAD: Computer-aided design, STL: Stereolithography, DICOM: Digital Imaging and Communications in Medicine.

Metal artifacts in DICOM images caused by dental metallic materials appear as imaging noise and consequently reduce the detail of the 3D model. For this reason, they are removed as much as possible during the creation of the STL model, but some may remain. We adjusted the position of the head and jaw and the tilt of the gantry to narrow the range of artifacts (specifically, the occlusal plane should be perpendicular to the floor during CT scanning) (Figure 9). Additionally, we also performed scanning with the patient's mouth kept slightly open using a bite block at a level to facilitate the segmentation of the maxilla and mandible on the STL model. Sometimes, the quality of the source data is not suitable for 3D printing. In many cases, the images are exported in thick slices (Figure 10). If the time has passed since scanning and no CT raw data have been stored (the values of all detector signals are measured during the scanning; from these data, a CT image is reconstructed by mathematical methods; the raw data can be used later for additional planes and images reconstruction; due to the size of the data, it is usually stored on a server for a few weeks and then deleted due to storage capacity limitations), the image cannot be reconstructed and re-exported with a thinner slice thickness. Just as companies that provide surgical guides and stents for dental implant surgery provide detailed instructions during CT scanning and DICOM file exporting, they should provide appropriate instructions during 3D printing.

Desktop FDM 3D printers, even if they are simply "personal" or "low-end," have significant advantages in their economics [7,17,18]. The "one-stop" 3D printing solution, where the whole process from STL model creation to 3D printing can be completed in-house, offers many advantages. Over the last seven years, our team has used three FDM 3D printers [6,19,20], and each one represented an improvement on the previous generation of printers, with stability continuing to increase. Unfortunately, however, experience has shown that the printing stability of FDM 3D printers is still not good enough, and many solutions must be mastered for successful fabrication. We have discussed that there are many variations in 3D printing failures. We have suggested a solution to avoid these. In our experience, the success rate of 3D printing is about 75%, i.e., the 3D printer loads the G-code data and finishes building without encountering any problems. Since there is no precedent for this, it is difficult to judge whether this value is high or low, and this is just our opinion. However, this means that FDM 3D printing is not an easy process.

It is very difficult to predict the future, but we expect 3D printing technology to continue to evolve and the sophistication and complexity of the data required to increase. Knowledge and techniques related to 3D printing technology will become increasingly important in the future. While it may be difficult to unify opinions on the optimal CT scan parameters and 3D printing parameters, as they vary from patient to patient and facility to facility, consolidating measures to reduce 3D printing failures will be essential for the future use of 3D models in medicine. It is expected that a system with artificial intelligence will solve these problems, such as automatically detecting and setting various optimal parameters without human intervention.

## Conclusions

It is no exaggeration to say that the introduction of 3D printing technology in the field of oral and maxillofacial surgery can provide clinical benefits to all. From an economic standpoint, the FDM 3D printer in particular could play an important role. As the market for 3D printers, not just FDM 3D printers, grows and is used for various applications, knowledge of 3D imaging engineering and 3D printing technology will become increasingly important. This knowledge will be even more essential if surgeons are to fabricate their 3D models. It is hoped that this report will help operators that are not 3D printing experts understand what to learn and how to use 3D printing technology to create 3D models efficiently and without failure.

## References

[1] Santler G, Karcher H, Ruda C (1998). Indications and limitations of three-dimensional models in cranio-maxillofacial surgery. J Craniomaxillofac Surg.

[2] Asaumi J, Kawai N, Honda Y, Shigehara H, Wakasa T, Kishi K (2001). Comparison of three-dimensional computed tomography with rapid prototype models in the management of coronoid hyperplasia. Dentomaxillofac Radiol.

[3] Dhanda J, Opie N, Webster K, Tanday A, Mumtaz S, Visram S (2011). Impact of modernising medical careers on basic surgical training and experience of oral and maxillofacial higher surgical trainees. Br J Oral Maxillofac Surg.

[4] McMenamin PG, Quayle MR, McHenry CR, Adams JW (2014). The production of anatomical teaching resources using three-dimensional (3D) printing technology. Anat Sci Educ.

[5] Seifert LB, Schnurr B, Herrera‐Vizcaino C (2020). 3D‐printed patient individualised models vs cadaveric models in an undergraduate oral and maxillofacial surgery curriculum: comparison of student's perceptions. Eur J Dent Educ.

[6] Kamio T, Hayashi K, Onda T (2018). Utilizing a low-cost desktop 3D printer to develop a "one-stop 3D printing lab" for oral and maxillofacial surgery and dentistry fields. 3D Print Med.

[7] Narita M, Takaki T, Shibahara T, Iwamoto M, Yakushiji T, Kamio T (2020). Utilization of desktop 3D printer-fabricated "cost-effective" 3D models in orthognathic surgery. Maxillofac Plast Reconstr Surg.

[8] Cohen A, Laviv A, Berman P, Nashef R, Abu-Tair J (2009). Mandibular reconstruction using stereolithographic 3-dimensional printing modeling technology. Oral Surg Oral Med Oral Pathol Oral Radiol Endod.

[9] Sotsuka Y, Nishimoto S (2014). Making three-dimensional mandible models using a personal three-dimensional printer. J Plast Reconstr Aesthet Surg.

[10] (2022). ISO/ASTM 52900:2021 additive manufacturing-general principles-terminology-fundamentals and vocabulary. http://www.iso.org/standard/74514.html.

[11] Malik HH, Darwood AR, Shaunak S, Kulatilake P, El-Hilly AA, Mulki O, Baskaradas A (2015). Three-dimensional printing in surgery: a review of current surgical applications. J Surg Res.

[12] Pabst A, Goetze E, Thiem DG (2022). 3D printing in oral and maxillofacial surgery: a nationwide survey among university and non-university hospitals and private practices in Germany. Clin Oral Investig.

[13] George E, Liacouras P, Rybicki FJ, Mitsouras D (2017). Measuring and establishing the accuracy and reproducibility of 3D printed medical models. Radiographics.

[14] Kamio T, Suzuki M, Asaumi R, Kawai T (2020). DICOM segmentation and STL creation for 3D printing: a process and software package comparison for osseous anatomy. 3D Print Med.

[15] Mitsouras D, Liacouras P, Imanzadeh A (2015). Medical 3D printing for the radiologist. Radiographics.

[16] Bhavsar P, Sharma B, Moscoso-Kingsley W (2020). Detecting first layer bond quality during FDM 3D printing using a discrete wavelet energy approach. Procedia Manuf.

[17] Choonara YE, du Toit LC, Kumar P, Kondiah PP, Pillay V (2016). 3D-printing and the effect on medical costs: a new era?. Expert Rev Pharmacoecon Outcomes Res.

[18] McAllister P, Watson M, Burke E (2018). A cost-effective, in-house, positioning and cutting guide system for orthognathic surgery. J Maxillofac Oral Surg.

[19] Kato H, Kamio T (2015). Diagnosis and endodontic management of fused mandibular second molar and paramolar with concrescent supernumerary tooth using cone-beam CT and 3-D printing technology: a case report. Bull Tokyo Dent Coll.

[20] Kamio T, Kato H (2019). Autotransplantation of impacted third molar using 3D printing technology: a case report. Bull Tokyo Dent Coll.

